# Oncolytic Virotherapy in Solid Tumors: The Challenges and Achievements

**DOI:** 10.3390/cancers13040588

**Published:** 2021-02-03

**Authors:** Ke-Tao Jin, Wen-Lin Du, Yu-Yao Liu, Huan-Rong Lan, Jing-Xing Si, Xiao-Zhou Mou

**Affiliations:** 1 Department of Colorectal Surgery, Affiliated Jinhua Hospital, Zhejiang University School of Medicine, Jinhua 321000, China; jinketao2001@zju.edu.cn (K.-T.J.); lyy21918555@zju.edu.cn (Y.-Y.L.); 2 Key Laboratory of Gastroenterology of Zhejiang Province, Zhejiang Provincial People’s Hospital, People’s Hospital of Hangzhou Medical College, Hangzhou 310014, China; dwl1209@163.com; 3 Clinical Research Institute, Zhejiang Provincial People’s Hospital, People’s Hospital of Hangzhou Medical College, Hangzhou 310014, China; 4 Department of Breast and Thyroid Surgery, Affiliated Jinhua Hospital, Zhejiang University School of Medicine, Jinhua 321000, China; lanhr2018@163.com

**Keywords:** oncolytic virus, tumor microenvironment, antitumor immune response, delivery, genetic modification

## Abstract

**Simple Summary:**

Oncolytic virotherapy (OVT) is a promising approach in cancer immunotherapy. Oncolytic viruses (OVs) could be applied in cancer immunotherapy without in-depth knowledge of tumor antigens. Improving efficacy, employing immunostimulatory elements, changing the immunosuppressive tumor microenvironment (TME) to inflammatory TME, optimizing their delivery system, and increasing the safety are the main areas of OVs manipulations. Recently, the reciprocal interaction of OVs and TME has become a hot topic for investigators to enhance the efficacy of OVT with less off-target adverse events. Current investigations suggest that the main application of OVT is to provoke the antitumor immune response in the TME, which synergize the effects of other immunotherapies such as immune-checkpoint blockers and adoptive cell therapy. In this review, we focused on the effects of OVs on the TME and antitumor immune responses. Furthermore, OVT challenges, including its moderate efficiency, safety concerns, and delivery strategies, along with recent achievements to overcome challenges, are thoroughly discussed.

**Abstract:**

Oncolytic virotherapy (OVT) is a promising approach in cancer immunotherapy. Oncolytic viruses (OVs) could be applied in cancer immunotherapy without in-depth knowledge of tumor antigens. The capability of genetic modification makes OVs exciting therapeutic tools with a high potential for manipulation. Improving efficacy, employing immunostimulatory elements, changing the immunosuppressive tumor microenvironment (TME) to inflammatory TME, optimizing their delivery system, and increasing the safety are the main areas of OVs manipulations. Recently, the reciprocal interaction of OVs and TME has become a hot topic for investigators to enhance the efficacy of OVT with less off-target adverse events. Current investigations suggest that the main application of OVT is to provoke the antitumor immune response in the TME, which synergize the effects of other immunotherapies such as immune-checkpoint blockers and adoptive cell therapy. In this review, we focused on the effects of OVs on the TME and antitumor immune responses. Furthermore, OVT challenges, including its moderate efficiency, safety concerns, and delivery strategies, along with recent achievements to overcome challenges, are thoroughly discussed.

## 1. Introduction

The first hints of the possible anticancer effects of viruses occurred during the early 20th century, with evidence of tumor regression in patients with simultaneous viral infections [1]. Such reports persisted until the 1950s, when the primary clinical studies on the tumor-killing ability of viruses that form the cornerstone of today’s achievements were carried-out [2]. Since then, various preclinical and clinical studies have attempted to optimize the viruses for increasing specificity, efficiency, and reducing adverse events (AEs), which led to the introduction of oncolytic virotherapy (OVT) as emerging immunotherapy of cancers [3]. Oncolytic virus (OVs) or cancer-killing viruses are defined as natural or genetically modified viruses that are able to selectively proliferate in tumor cells without damaging normal cells [4]. This natural tropism of some viruses to tumors is due to an increase in some receptors (such as CD54) on the surfaces of tumor cells or defects of tumor cells to induce innate immunity against viruses [5]. So far, various DNA and RNA OVs have been used to treat cancer [6]. The majority of DNA viruses are double-stranded, while RNA viruses are predominantly single-stranded. The advantages of double-stranded DNA viruses are their large genomes which enable them to carry large eukaryotic transgenes and high fidelity DNA polymerase, maintaining the virus genome integrity during replication [7]. Regarding their relatively small size, RNA viruses cannot encode large transgenes. However, they are better candidates in the delivery system due to less induction of immune responses [8]. Several RNA viruses and DNA viruses, including reovirus (RV), Seneca Valley virus (SVV), poliovirus (PoV), parvovirus (PV), vaccinia virus (VACV), and herpes simplex virus (HSV) have the ability to cross the blood-brain barrier (BBB) enabling their use in brain tumors [9,10,11,12,13,14]. OVT started with wild-type viruses such as Newcastle disease virus (NDV), myxoma virus (MYXV), SVV, PV, coxsackievirus (CV), and RV [3]. However, genetic modification was a revolutionary achievement in the OVT providing greater specificity and efficacy against tumors with higher safety for healthy cells [15]. Genetically modified OVs (GMOVs) mainly include PoV, measles virus (MeV), adenovirus (AdV), VACV, HSV, and vesicular stomatitis virus (VSV) [3]. The first GMOV was HSV-1, introduced in 1991 [16]. So far, three OV-based drugs have been approved for cancer treatment, the first of which was an unmodified ECHO-7 virus called Rigavirus which was approved in 2004 in Lativa under the brand name Rigvir for melanoma [17]. However, the approval was withdrawn in 2019 due to its low efficacy. The two other approved OVs are GMOVs include Oncorine (H101 adenovirus), which obtained approval for head and neck cancer in China in 2005 [3], and T-VEC or Imlygic (HSV-1), which was approved in 2015 in the United States and Europe for non-surgical melanoma [18]. The efficacy of OVs on many cancers, such as melanoma, glioblastoma, triple-negative breast cancer (TNBC), head and neck cancers, and colorectal cancers has been elucidated [19,20,21,22,23], and a large number of clinical trials are currently evaluating the wild-type and GMOVs efficiency and safety in various cancers which are listed in Table 1. Along with the therapeutic approaches, GMOVs expressing reporter genes can be applied in the diagnosis of various cancers by positron emission tomography or single-photon emission computed tomography [24].

OVs can kill the tumor cells in the following main ways: 1. OVs infect and replicate specifically in tumor cells leading to direct lysis of tumor cells. Malignant cells have defects in antiviral responses allowing OVs to replicate and lyse malignant cells [7]; 2. OVs can induce different types of immunogenic cell death (ICD), including necrosis, necroptosis, immunologic apoptosis, pyroptosis, and autophagy. Tumor cell death or lysis causes the release of tumor-associated antigens (TAA) and neoantigens (TAN) and damage-associated molecular patterns (DAMPs), which increase inflammation and improve the efficacy of immunotherapy [25,26]; 3. OVs, especially GMOVs, can enhance tumor antigen presentation and prime the immune response in the tumor microenvironment (TME) by induction of antiviral responses, inflammation, cytokine production, and expression of costimulatory molecules [26,27]; 4. The infection of vascular endothelial cells (vECs) by OVs destroys tumor vasculature, resulting in tumor necrosis and the infiltration of immune cells into the TME [28].

Accordingly, a considerable part of OVT effects on tumors is achieved by changing the TME from an immunosuppressive to the immunostimulatory microenvironment and affecting the tumor vasculature and matrix. Moreover, the success of OVT in solid tumors largely depends on the OV access to the tumor. Here, we review the effects of OVs on the TME and antitumor immune responses. Furthermore, OVT challenges, including its moderate efficiency and safety concerns, along with recent achievements to overcome challenges, are thoroughly discussed. Regarding the critical role of OV delivery strategy in the efficacy of OVT, recent approaches enhancing OV delivery into the TME are also provided.

## 2. Oncolytic Virus Effects on TME

The long-term effects of immunotherapy in solid tumors are mostly unsatisfactory, partly due to the immunosuppressive condition of TME and low infiltration of immune cells. TME consists of tumor cells, tumor-associated fibroblasts (TAF), vEC, mesenchymal cells, myeloid-derived suppressor cells (MDSCs), and tumor-infiltrating leukocytes (TILs), such as T cells, B cells, dendritic cells (DCs), natural killer (NK) cells, macrophages, and neutrophils [90]. The presence of exhausted cytotoxic T lymphocytes (CTLs), helper T-cells (THs), and NK cells, as well as a large number of regulatory T-cells (Tregs), tolerogenic DCs, MDSC, and M2-macrophages, induce immunosuppressive milieu in the TME through inhibitory ligands and secretion of inhibitory cytokines such as interleukin (IL)-10, tumor growth factor (TGF)-β, IL-35, and IL-27 [91]. OVs can change the paradigm in the TME and convert cold tumors to hot ones by various mechanisms.

### 2.1. OV-Mediated Lysis of Tumor

Direct oncolysis activity of OVs is the first stimulus of the immune response in the TME [92]. Overexpression of surface receptors such as CD46, CD54, CD155, CD55, and integrins enhances OVs’ preferable entry to tumor cells [93,94,95,96,97]. In normal cells, viral components known as pathogen-associated molecular patterns (PAMPs) are sensed by pattern recognition receptors (PRRs) and induce the production of interferon (IFN)-I through the Janus kinase signal transducer and activator of transcription (JAK-STAT) and Nuclear Factor (NF)-kB signaling pathways. IFN-I activates the protein kinase RNA-activated (PKR) signaling pathway leading to protein synthesis blockade and viral clearance [98]. Tumor cells have defects in antiviral pathways such as IFN-I, PKR, and JAK-STAT, resulting in the survival and proliferation of OVs, specifically in tumor cells [99,100,101]. Lysis of OV-infected cells releases a very diverse TAAs that prime immune cells to induce a local and systemic vaccination against the released TAAs [92]. While many cancer immunotherapies depend on identifying and targeting TAAs (one or several limited TAAs), OVT can vaccinate patients against the entire TAA and TAN treasure of cancer through a phenomenon called antigen/epitope spreading. Hence, OVT could be considered a kind of personalized immunotherapy. Interestingly enough, recent studies have reported the increase of TAA- and TAN-specific T cells in the blood of patients with melanoma and ovarian cancer treated with OVs, suggesting that the in situ OV injection might enhance the systemic antitumor response [102,103,104]. This finding raises hopes for the anti-metastatic effects of OVT. TANs are assumed to be derived from high mutational burden of tumor cells [105,106]. These immunogenic TANs are capable of eliciting tumor-specific immune responses and serve as ideal targets in immunotherapy [105,106,107]. However, TAN-specific T cells are not activated enough in cancer patients due to the poor presentation of TANs, lack of costimulatory signals, and abundance of inhibitory immune checkpoints in the TME [107]. OVs, especially armed OVs, have been shown to activate the TANs-specific T cells by increasing the access of APCs to the TANs (epitope spreading), enhancing the TANs processing and presentation by APCs, and providing costimulatory signals [107,108,109]. Accordingly, Wang et al. demonstrated that VACV armed with PD-L1 inhibitor and GM-CSF enhanced TANs presentation and activated systemic T cell responses against dominant and subdominant (cryptic) neoantigens [107], so OVT could potentiate the antitumor immune responses by activating the TANs-specific T cells.

### 2.2. Induction of Immunologic Cell Death

Apart from the direct lysis of cancer cells, OVs can induce various ICDs in virus-infected cells through induction of endoplasmic reticulum (ER) stress [110]. Infection of tumor cells with AdV, CV-B3, MeV, VACV, HSV, and H1-PV has been shown to induce ICD and autophagy in cancer cells [111,112]. ICD is characterized by the expression and release of DAMPs such as ATP, uric acid, heat shock proteins, ecto-calreticulin, and HMGB1, as well as extracellular proinflammatory cytokines [113]. Extracellular ATP acts as a danger signal which attracts and activates DCs [114]. HMGB1 and calreticulin can activate DCs via toll-like receptor (TLR)-4 signaling [115]. In addition, calreticulin neutralizes CD47 receptors on the tumor cell surface, and thereby, increases the tumor cell engulfment by macrophages [116]. OV-mediated ICD, along with other ICD-inducing methods such as chemotherapy and radiotherapy, break immune tolerance against the tumor and increase lymphocyte and neutrophil infiltration, leading to antitumor response and more survival in preclinical models [111].

### 2.3. Stimulation of Antitumor Immune Response

Besides the release of DAMPs, cancer cell death also causes the release of viral PAMPs in the TME. These PAMPs mainly include DNA, ssRNA, dsRNA, proteins, and capsid contents that activate innate immune cells through stimulating PRRs such as retinoic acid-inducible gene (RIG)-1, cyclic GMP-AMP synthase (cGAS), and stimulator of interferon genes (STING) [113]. DCs, as a bridge between the innate and adaptive immune systems, play a critical role in generating the antitumor response. DCs elicit a specific response against TAA-expressing tumor cells by engulfing OV-infected cells and cross-presentation of TAAs to CD8+ T and CD4+ T cells [117]. On the other hand, the OVs-derived PAMPs cause maturation of myeloid and plasmacytoid DCs, leading to the production of proinflammatory cytokines such as IFN-α, IFN-γ, IL-12, IL-1β, IL-6, IL-8, and tumor necrosis factor (TNF)-α [90,118,119]. These functional DCs, mainly CD103+ and BATF3+, prime CD8+ T cells against tumors [120]. Innate immune signaling, such as the cGAS-STING pathway, plays a pivotal role in the recruitment of lymphocytes to the TME through the expression of CXCL9 and CXCL10 [121]. Parallel to DCs, innate lymphoid cells (ILCs) also respond to the released PAMPs leading to higher inflammation and antitumor responses [18]. As an example, arenavirus-infected melanoma cells produce a high level of CCL5, leading to recruitment of NK cells and melanoma regression [122]. Interestingly, in situ antitumor responses following OVT are mainly mediated by IFN-I, whereas OVT-mediated systemic antitumor responses appear to be mediated by IFN-II excreted from TILs [123]. In general, the innate immune response to OVs increases lymphocyte infiltration, antigen presentation, and activation of the antitumor adaptive immune response through an IFN-mediated mechanism [18]. T cell activation requires at least three consecutive signals (peptide-MHC, CD28-B7, and stimulatory cytokines), all of which are defected in TME to escape adaptive immune responses. OVs, as potent immunogens, induce all three signals needed to activate T cells [18]. OVT increases the expression of B7-1/2 and CD40 on the surface of DCs and induces the expression of MHC-peptide on the surface of tumor cells leading to optimal activation of T cells [124]. Conversion of the TME phenotype from immunologically inert to immunologically active status can augment the effectiveness of the immunotherapeutic modalities.

### 2.4. Effect of OV on Tumor Vasculature

Some OVs, such as HSVs and VACVs, can target tumor stromal cells, such as TAFs, vECs, and pericytes, thereby destroy the tumor’s complex structure [26]. TGF-β secreted by tumor cells makes TAFs susceptible to OV infection [125]. OVs also reduce the fibrosis in the TME. VSV has been shown to infect hepatic stellate cells (HSCs), leading to tumor fibrosis reduction [126]. OVs affect the tumors vasculature by replicating in the tumor vECs. Vascular endothelial growth factor (VEGF) secreted from tumor vECs suppresses the antiviral response and allows the replication of OVs in endothelial cells through ERK1/2 and STAT3 pathways [127]. Following infection and replication, the OVs reduce VEGF production from the infected cell resulting in angiogenesis prevention in the tumor. OVs’ antiangiogenic properties further limit tumor growth by decreasing the oxygen and nutrition supplies [6]. VACV is shown to replicate in the tumor vEC and cause vascular destruction and ischemia [28]. Neutrophil infiltration into the TME seems essential for OVT-mediated ischemia through the induction of thrombosis in small tumor vessels [28]. It has been shown that the administration of JX-594 in hepatocellular carcinoma destroyed tumor vasculature without affecting patients’ normal vessels [28]. Thus, targeting of stromal cells by OVs increases the infiltration of immune cells into the TME, and converts immuno-deserted or immune-excluded tumors (with low TILs) into immune-infiltrated tumors [18]. OVT-mediated changes in the TME, including lymphocyte infiltration into the tumor, enhancement of TAAs/TANs presentation, and heating the TME can improve other immunotherapies such as adoptive cell therapy (ACT) and immune checkpoint inhibitors (ICIs) [90].

## 3. OVT Challenges and Achievements

### 3.1. Tumor Targeting 

Although OVs have tumor tropism based on some overexpressed receptors and adhesion molecules on the tumor cells, the tumor tropism of wild OVs is not enough. GMOVs can express receptors with a high affinity for TAAs. For instance, insertion of single-chain antibodies (scAb) against human epidermal growth factor receptor (HER)-2, epithelial cell adhesion molecule (EpCAM), and carcinoembryonic antigen (CEA) increases the specificity of OVs to tumors [128,129,130]. Insertion of sequences such as the arginine-glycine-aspartate (RGD) motif or specific domain from AdV3 and AdV35 to AdV5, makes AdV5 specific for integrins, desmoglein-2, and CD46, which are overexpressed in tumors [131,132]. VSV expressing HIV-derived glycoprotein (gp)-160 is a specific VSV against leukemia and T lymphomas [133].

Defects in the IFN-I antiviral response, lack of tumor suppressor genes such as the retinoblastoma (Rb), and increased Ras signaling in tumor cells lead to the specific proliferation of OVs in tumor cells [134]. Insertion of tumor-specific promoters such as prostate-specific antigen (PSA) and human telomerase reverse transcriptase (hTERT) promoters, which are highly expressed in tumor cells, causes specific expression of viral genes in tumor cells [135,136]. Some micro-RNAs (miRNAs) are overexpressed in healthy cells while they are at negligible levels in tumor cells. Hence, targeting these miRNAs by miRNA-targeting sequences (miRNA-TS) destroys viral RNA in normal cells. Low expression of miRNA-TS targets in tumor cells causes viral RNAs to remain and replicate in tumor cells [137].

### 3.2. Improving Antitumor Efficacy

**Figure 1:**
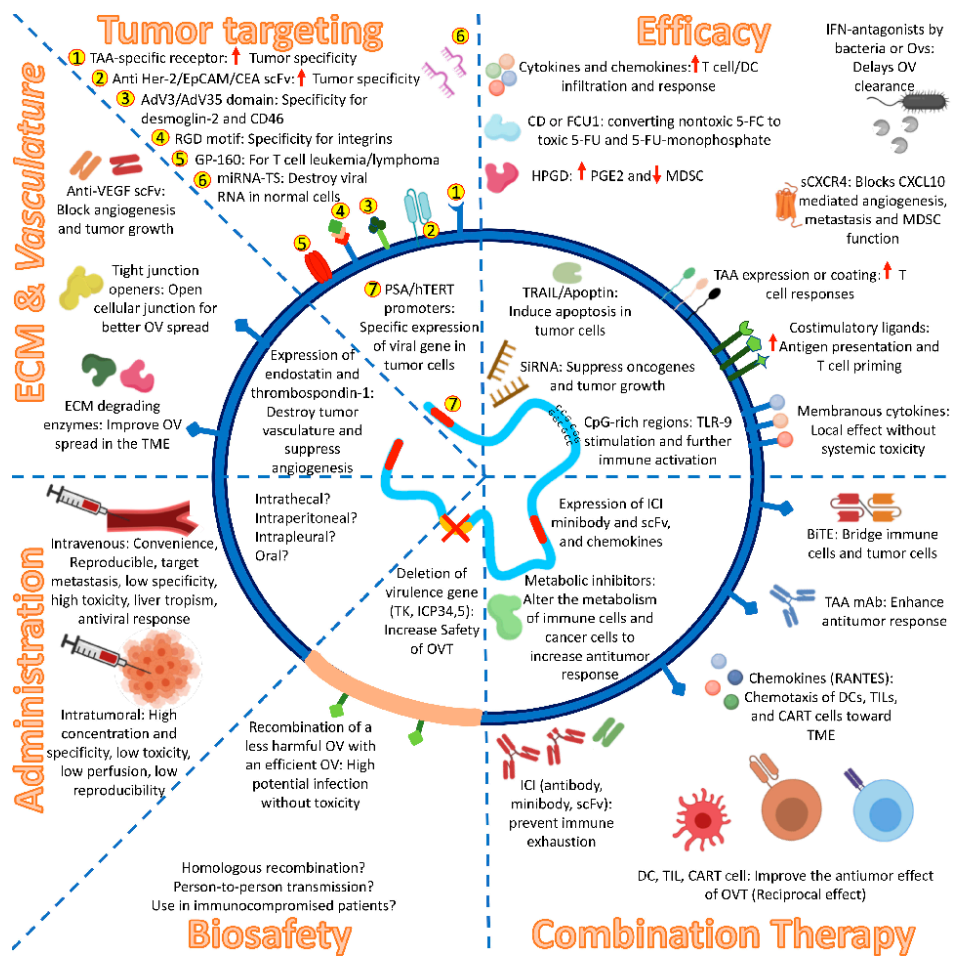
Recent approaches in oncolytic virotherapy. Expression of TAA-receptors and scFvs, recombination of specific domains and motifs, using tumor-specific promoters, and application of miRNA-TS could enhance the tumor targeting. GMOVs expressed inflammatory cytokines, enzymes, chemokine receptors, costimulatory molecules, and proapoptotic proteins achieve high antitumor potency. ECM and vasculature degradation by enzymes and molecules result in a higher spread of OVs. Administration routs are a critical factor in achieving better results with lower adverse effects. Besides, deleting virulence genes and recombination of OVs together could diminish the concerns of adverse events. However, several biosafety concerns still remained unmet. The combination of OVT with other immunotherapy, such as ICIs, TIL therapy, CART cell therapy, DC vaccines, mAbs, BiTEs, and metabolic inhibitors could potentiate the immunotherapy against tumors. OV. Oncolytic virus; OVT. OV therapy; TAA. Tumor-associated antigen; scFv. Single-chain variable fragment; Her-2. Human epidermal growth factor receptor 2; EpCAM. Epithelial cell adhesion molecule; CEA. Carcinoembryonic antigen; AdV. Adenovirus; RGD. Arginine-glycine-aspartate; GP-160. Glycoprotein-160; miRNA-TS. microRNA targeting sequence; PSA. Prostate-specific antigen; hTERT. Human telomerase reverse transcriptase; DC. Dendritic cell; CD. Cytosine deaminase; 5-FC. 5-fuorocytosine; 5-FU. 5-fluorouracil; HPGD. Hydroxyprostaglandin dehydrogenase; PGE2. Prostagalndin-E2; MDSC. Myeloid-derived suppressor cell; IFN. Interferon; TRAIL. TNF-related apoptosis-inducing ligand; siRNA. Small interfering RNA; TLR-9. Toll-like receptor-9; ICI. Immune checkpoint inhibitor; BiTE. Bispecific T cell engager; mAb. Monoclonal antibody; TIL. Tumor0infiltraring lymphocyte; CART cell. Chimeric-antigen receptor T cell; TME. Tumor microenvironment; ECM. Extracellular matrix; VEGF. Vascular-endothelial growth factor.

Genetic modifications of OVs to increase the expression of cytokines, chemokines, costimulatory molecules, tumor extracellular matrix (ECM)-degrading enzymes, and antiangiogenic molecules can enhance their antitumor effects (Figure 1). Granulocyte-macrophage colony-stimulating factor (GM-CSF) gene-bearing OVs such as T-VEC, Pexa-Vec, and CG0070 recruit antigen-presenting cells (APCs) and CTLs, resulting in a better TAA presentation with minimal antiviral response induction [6]. GMOVs expressing proinflammatory cytokines showed enhanced antitumor efficacy. Despite the considerable antitumor response, IL-2-secreting OVs cause systemic toxicity. The design of VACV expressing membranous IL-2 rather than secretory form increases local antitumor response with significantly reduced toxicity [138]. The use of IL-12, IL-15, IL-18, TNF-α, IL-24, and IFN-γ genes in OVs also enhances antitumor effects with much lower toxicity than IL-2 [6,139,140,141]. Interestingly, the application of the non-secretory form of these cytokines causes local effects rather than systemic AEs [142]. Expression of specific chemokines such as CCL5, CCL19, CCL20, CCL21 by engineered OVs (mainly VACV) increases the infiltration of naïve and memory T lymphocytes and DCs into the TME [143,144,145,146]. Simultaneously, employment of one or multiple costimulatory ligands, including CD40L, 4-1BBL, OX40L, and B7-1 in OVs such as LOAd703 (the combination of CD40L and 4-1BBL) increases antigen presentation and T cell priming [6,26,96]. Besides, insertion of TLR ligands such as CpG-rich regions in the OVs genome stimulates TLRs and further activates innate and acquired immunity [138].

Another way to enhance the immune responses in the TME is the elimination of immunosuppressive cells. GMOVs that express the hydroxyprostaglandin dehydrogenase (HPGD) enzyme inactivate PGE2 and reduce the presence of MDSCs in the TME [147]. Soluble CXCR4 expressed by GMOVs binds to CXCL12 secreted by tumor cells as a decoy receptor and inhibits the effects of CXCL12 on angiogenesis, metastasis, and recruitment of MDSCs [148]. 

Although OVT can release TAAs through various mechanisms, the expression of TAAs by GMOVs or coating the TAA-derived peptides on the surface of OVs increases T cell response and improves OVT. A large number of TAAs and peptides have been studied so far [26]. The advantage of peptide coating over peptide expression is the convenience, speed, lower cost, and the possibility of personalization for each patient in the peptide coating method [26].

OVs can be engineered to express proapoptotic proteins such as TNF-related apoptosis-inducing ligand (TRAIL) and apoptin that can induce specific apoptosis in tumor cells [149,150]. Insertion of the oncogene suppressor small interfering RNAs (siRNAs) in OVs could also suppress oncogene expression and inhibit tumor growth [151,152]. 

The host antiviral response ensures that OVs disappear after a while and prevents the AEs of their long presence. However, the host antiviral response might cause rapid clearance of OVs before fulfilling their antitumor activity [153]. Expression of IFN-I antagonists by OVs or some non-pathogenic bacteria reduces the innate immune response against OV and delays their clearance [154]. Also, the use of stem cells, polymers, and liposomes as OV carriers reduces the immunogenicity of OVs, shields them from neutralizing antibodies (nAbs), and improves their transmission to the TME, which is listed in Table 2. An interesting way to optimize cytokine production with minimal antiviral responses is to insert inducible promoters or regulatory genes so that the cytokine expression is exogenously induced after sufficient replication of OVs in tumor cells [155].

### 3.3. Tumor ECM and Vasculature Degradation

Tumor ECM is a barrier to access tumor cells. Co-administration of ECM-degrading enzymes such as relaxin [234], matrix metalloproteinase (MMP)-1, -8, -9 [131,226], chondroitinase [235], and hyaluronidase [226] with OVT, or induction of their genes expression in GLV-1h255 (VACV) and VCN-01 (OAdV) can increase OV spread into the TME and improved OVT efficiency in cancers such as retinoblastoma and pancreatic carcinoma [236,237]. Cellular tight junctions are also accounted as barriers for OV distribution. GMOVs can trigger the production of proteins such as penton-dodecahedra and junction opener-1, which open the cellular junction through binding to desmoglein-2 [226]. However, there are concerns about increasing the likelihood of metastasis in this method that needs further investigation. 

On the other hand, the insertion of endostatin and thrombospondin-1 genes in HSV-Endo and T-TSP-1 (both are HSV) destroys tumor vasculature. It suppresses angiogenesis in lung and gastric cancer by inhibiting migration and enhancing apoptosis in vECs [238,239]. Also, the expression of anti-VEGF sc-Ab by VACV increases antiangiogenic and antitumor properties [240].

### 3.4. Biosafety of OVT

Besides tumor cells, some OVs might replicate in normal cells and cause damage. For instance, T-VEC might remain a latent infection and cause long-term neurological AEs [153]. Using OVs with low pathogenicity in humans, such as parvovirus and reovirus, weakening OVs through repeated passages or deleting virulence genes, can increase the safety of OVT [241,242]. Thymidine kinase (TK) and infected cell protein (ICP)34.5 genes play a vital role in VACV and HSV-1 replication. The products of such genes are abundant in tumor cells, so the GMOVs lacking these genes can replicate in tumor cells, while the virus replication is impaired in healthy cells due to the low expression of such products [243,244]. The GL-ONC1 and Pexa-Vec (JX-594) are TK-free VACVs, and the T-VEC, HSV-1716, and G207 are ICP34.5-free HSVs showing acceptable safety in clinical trials [6,245,246,247]. Wild ZIKA virus has oncolytic potential in glioblastoma but also infects normal nerves with severe complications. Removal of 10 nucleotides from 3’ of its genome can increase safety without reducing oncolytic activity [248]. Mutation or deletion of the E1 gene in AdV, and deletion of TK, vaccinia growth factor (VGF), hemagglutinin, and B18R genes in poxvirus reduce the virulence of OVs in normal cells [153,249]. However, deleting virulence genes to increase safety sometimes reduces OVs’ antitumor activity [250].

Recombination of a safe OV such as NDV with an efficient OV like VSV is another way to increase the safety of OVs. Recombinant VSV-NDV (rVSV-NDV) comprises the envelope contents from NDV and the original backbone of VSV. Recombination of AdV with less harmful coxsackievirus or parvovirus constitutes OVs with high potency in tumor cell infection without damage to normal cells [250,251,252]. Using Ebolavirus (EBOV) glycoproteins also reduces the neurotoxicity of VSV in rVSV-EBOV [253]. Nevertheless, naturally occurring homologous recombination of GMOVs and wild-type OVs might result in a transgenic and pathogenic virus [153]. Transmission of OVs through body fluids to other people is rare but still a concern [254]. Also, the safety of OVT in immunocompromised individuals receiving radiotherapy and chemotherapy, as well as in pregnant women is still debated [153]. In general, due to the emergence of OVT with GMOVs, its long-term AEs are still unknown and require caution and further investigations.

### 3.5. Administration Routs

One of the factors influencing the response to OVT is the way of administration. Intratumoral injection results in precise control of the OV concentration in the TME, resulting in better therapeutic outcomes [255,256]. However, the complexity of intratumoral injection limits dosing repetition [257]. Besides, low perfusion of OVs into dense tumors requires ECM-degradation strategies [226]. Intravenous injection is popular due to its convenience, reproducibility, and possibility to target metastatic foci [258,259]. However, it requires tumor- specific delivery systems and is more likely to cause systemic toxicity [257]. Liver tropism, physical barriers such as BBB, complement activation, and the immune system response to OV before accessing the TME are the other disadvantages of intravenous injection [226,257]. Intraperitoneal, intrathecal/intracranial, and intrapleural injections are suitable for targeting intra-abdominal organs, central nervous system (CNS), and lung tumors, respectively, but are limited to use in laboratory animals [153]. The best route of administration is still a matter of debate with no specific guidelines. It seems that the less aggressive administration routs such as oral/mucosal and nasal administration, at least for gastrointestinal and cerebral malignancies, could increase the acceptability for patients and should be considered in future studies.

## 4. Combination Therapy

### 4.1. Immune-Checkpoint and Cell Therapy

Despite all the benefits, OVT as monotherapy cannot have a dramatic effect on tumor suppression and, like other immunotherapy methods, is used as combination therapy. A common complementary treatment strategy for OVT is ICI [260]. The overexpression of various immune checkpoints in the TME suppresses the response of immune cells. OVT and ICI seem to have synergistic effects [114,260]. OVT facilitates the infiltration of immune cells into the TME, and ICIs prevent the suppression of infiltrated immune cells activity. OVT also improves ICI access to the TME by destroying ECM and tumor vessels [6]. Recently, the use of OVs expressing mini-antibody (minibody) and single-chain variable fragment (scFv) against checkpoints has been able to block checkpoints locally in the TME, with fewer AEs [261,262]. Many clinical trials are currently examining the combination of ICI and OVT, the results of which primarily suggest that in order to achieve a better outcome, ICI should be prescribed after the onset of OV responses [6,263]. OVT increases the effectiveness of TIL and CAR-T cell therapy. OVT can increase the access of TILs and CAR-T cells to the tumor by altering the tumor matrix and increasing the chemokines such as CCL5 [264]. The secretion of IL-15, TNF-α and IL-2 from OVs in the TME increase the in situ proliferation and activation of TILs and enhances tumor response to CAR-T cell therapy [265,266]. Bispecific T-cell engagers (BiTEs) are fusion proteins containing two scAbs against tumor antigens and T cell surface CD3 [267]. The use of BiTE-expressing OVs can bridge T/CAR-T cells to TAA-expressing cells in the TME [267]. Furthermore, concomitant use of TAA-specific mAbs with OVT can enhance the antitumor response. However, the small size of OVs genome has made it difficult to encode whole antibodies [268] Combination of OVT with DC vaccines also improves the efficacy of DC vaccines by altering the TME immunosuppressive conditions [269]. OVs could be utilized as tumor vaccines in order to enhance the immune responses against established tumors or even prevent tumor recurrence. The main function of such OV-based tumor vaccines is the recruitment of APCs, facilitating the phagocytosis of tumor cells by APCs, and promoting the APC maturation to induce appropriate antitumor immune responses [270,271,272].

### 4.2. Metabolic Inhibitors as an Emerging Combination Therapy

Given the OV dependence on host cell metabolism for replication, the metabolic pathways can be considered effective modalities in OVT. For example, due to the role of glycolysis in the antiviral response, blocking this pathway increases the sensitivity of cells to OV infection [273,274]. On the other hand, increasing pyruvate flux into the tricarboxylic acid cycle, the increment of oxidative phosphorylation, and reactive oxygen species production lead to enhance OV replication and oncolytic activity [275,276,277]. However, there are contradictions in the enhancing or dampening roles of these metabolic pathways in the replication and function of OVs [278]. These discrepancies indicate that the metabolic pathway targeting should be based on the type of cancer and employed OV. Tumor cells deplete the glucose, tryptophan, and glutamine required by immune cells and produce lactate, kynurenine, and adenosine [279]. These changes cause induction of exhausted CTLs, M2-macrophages, and Tregs, creating an immunosuppressive TME [275,276,277,278]. The combination of metabolic inhibitors with OVT and the application of GMOVs to express metabolic inhibitors can alter the metabolism of cancer cells and immune cells to increase antitumor responses [278,280].

### 4.3. Other Combination Therapies

Along with the growing interest in OVT in the field of cancer treatment, many preclinical and clinical studies have suggested the use of OVs in combination with other common cancer therapies. OVT has been shown to potentiate the response to chemotherapy and radiotherapy, so that it could re-sensitize the chemo-/radio-resistant cells. Therefore, the combination of OVT with chemotherapy and radiotherapy is currently being evaluated in several clinical trials for chemo-resistant patients (Table 1). One of the shared mechanisms of OVT and chemo-/radiotherapy is ICD, in which a plethora of DAMPs is released, resulting in maximum induction of innate and adaptive immune responses. Hence, using OVT along with chemo-/radiotherapy could decrease the required doses of toxic agents and consequently lessen the adverse events of high dose treatments. Recombinant OVs can express enzymes such as cytosine deaminase, which converts the non-toxic prodrug 5-fuorocytosine (5-FC) into a toxic drug 5-fluorouracil (5-FU) in the tumor milieu [152]. Such local production of chemotherapeutic agents would decrease the systemic adverse events. GMOVs encoding the FCU1 gene can produce two enzymes, FCY1 and FUR1, that convert 5-FC to 5-FU and consequently 5-FU-monophosphate to target 5-FU-resistant tumors [152]. The tumor ECM prevents the access of therapeutic agents to the tumor cells, making the tumor resistant to chemotherapy [281]. Combination of ECM-degrading GMOVs with chemotherapy overrides the ECM-induced chemo-resistance observed in solid tumors [281]. Combination therapy of OVs and chemotherapy has been shown to exert synergistic antitumor activities via enhancing tumor cell killing capacity of chemotherapeutic agents, increasing virus proliferation in tumor cells, and invigorating oncolytic activities of OVs [282,283].

Besides conventional chemotherapy and radiotherapy, OVs could be administered in combination with targeted therapies [284]. Histone deacetylase inhibitors (HDIs) are recently entered the clinic as a promising treatment for cancers [285]. The companion of HDIs with OVT increases viral replication, upregulates the transgene expression (such as GM-CSF in T-VEC), enhances virus spread through the tumor cells, and augments oncolytic activities [286,287]. Moreover, HDIs induce antitumor immunity by inducing the expression of NK cell activating ligands and expression of TAAs, resulting NK cells and CTLs priming [286]. Co-administration of OVs with some protein kinase inhibitors such as MEK-1/2 and BRAF, and also inhibitors of some transcription factors like STAT-1 and NK-κB has been shown to enhance the oncolytic activities of OVs [288,289]. MEK/BRAF inhibitors do not affect viral replication. Instead, they enhance ER stress-induced apoptosis following OVT [288]. Interferon-stimulated genes (ISGs) are associated with resistance of tumors to chemotherapy, radiotherapy, and OVT. STAT-1 and NK-κB inhibitors diminish the expression of ISGs and thereby increase the cytotoxicity of OVs [289].

## 5. Conclusions

Although the OVT is not a new concept in cancer, the concerns of possible adverse events and unspecific infection hamper enough development in this era. The emerging genetic manipulations of OVs facilitate clinical studies with much lower concerns and reintroduce OVT as a promising immunotherapeutic approach. However, many questions should still be addressed. Finding the appropriate OV for each tumor, the best combination therapy, higher OVT efficacy and safety, and optimal delivery system require further knowledge about the cellular and molecular interaction between the OVs and the cells present in the TME. The results of current clinical trials could pave the way for OVT in the clinic.

## Figures and Tables

**Table 1:** Oncolytic viruses that reached the clinical phase.

Oncolytic Virus	Modification	Combination Therapy	Cancer Type (Clinical Trial Phase)	Ref.
HSV-1	Virulence gene ICP34.5 and ICP47 are deleted and human GM-CSF gene is inserted	ICIs (anti-PD1, anti-CTLA4	Melanoma (I, II), Sarcoma (I, II)	[29,30,31]
		-	Breast cancer (I), Head and neck cancer (I, I/II), Gastrointestinal cancers (I), Melanoma (I, II, III)	[32,33,34,35,36]
	Virulence gene ICP34.5 is deleted	-	Oral SCC (I), Pediatric extracranial cancers (I)	[37,38]
		Chemotherapy	Chemo-resistant metastatic colon cancer (I, I/II)	[39,40]
	Virulence gene ICP34.5 is deleted and ICP6 gene is inactivated	Radiotherapy	Glioblastoma (I)	[41]
	Naturally mutated	-	Pancreatic cancer (I)	[42]
NDV	Autologous tumor lysate and IL-2 is added	-	Stage III of Melanoma (I)	[43]
	Naturally attenuated	-	Advanced solid tumors (I)	[44]
	One-cycle replicating cytopathogenic NDV	-	Glioblastoma (I/II)	[45]
CVA21	-	ICIs (anti-PD1)	NSCLC (Ib), Bladder cancer (Ib)	[46]
		-	Bladder cancer (II), Advanced melanoma (II)	[47,48]
RV	-	-	Advanced solid tumors (I), Recurrent glioma (I), Extracranial solid tumors (I), Melanoma (II), Pancreatic adenocarcinoma (II)	[49,50,51,52,53]
		Chemotherapy	Advanced solid tumors (I), Ovarian cancer (IIb), Peritoneal cancer (IIb), Melanoma (II), Metastatic breast cancer (II), Advanced head and neck cancer (I/II) Pancreatic adenocarcinoma (II)	[54,55,56,57,58]
		Radiotherapy	Advanced solid tumors (I)	[59]
PoV	Recombinant oral PoV Sabin-1: the internal ribosome entry site (IRES) is replaced with the IRES from human rhinovirus-2: nonpathogenic	-	Recurrent glioblastoma (I)	[60]
AdV	AdV3 fiber knob is inserted into the backbone of AdV5, A 24-base pair in the E1 gene is deleted: CRAd GM-CSF gene is inserted	-	Ovarian Cancer (I), Gynecologic malignancies (I), Advanced solid tumors (I)	[61,62,63]
		Chemotherapy	Chemo-resistant advanced solid tumors (I)	[61]
	RGD motif is inserted into the AdV5 fiber knob: Integrin targeted instead of CAR dependence GM-CSF gene is inserted	-	Chemo-resistant advanced solid tumors (I)	[64]
	Prostate-specific antigen (PSA)-selective	Radiotherapy	Metastatic prostate cancer (I)	[65]
	Conditionally replicating GM-CSF expressing AdV	-	Bladder Cancer (I, II), Head and neck cancers (I)	[66,67]
	Human telomerase reverse transcriptase (hTERT) is inserted: tumor selective replication	-	Advanced solid tumors (I)	[68]
	E1B-deleted AdV: selective replication in P53-deficient cells	Chemotherapy	Advanced solid tumors (I), Malignant glioma (I), Recurrent head and neck cancer (I, II), Gastrointestinal cancers (II), Colorectal cancer (II), Advanced sarcoma (I/II),	[69,70,71,72]
	Chimeric AdV: Ad11p/Ad3, AdV5- cytosine deaminase/HSV-1 thymidine kinase: suicide gene for safety	-	RCC (I), NSCLC (I), Colorectal cancer (I), Urothelial cancer (I),Prostate cancer (I, II), Glioma (II)	[73,74,75,76]
VACV	GM-CSF gene is inserted Thymidine kinase gene is deleted	Chemotherapy	Metastatic melanoma (I), HCC (I, II), Colorectal cancer (I), Ewing sarcoma (I), neuroblastoma (I),	[77,78,79,80]
	FCU1 transgene is inserted: metabolize 5-FC to 5-FU-monophosphate	Chemotherapy	Chemo-resistant liver tumors (I)	[81]
	Thymidine kinase gene and hemagglutinin gene and F14.5 gene are deleted Luciferase gene, beta-galactosidase, and beta-glucuronidase are inserted	Chemotherapy and radiotherapy	Head and neck cancer (I), Colorectal cancer (I) Advanced solid tumors (I)	[82,83,84]
MeV	Genetically modified to express carcinoembryonic antigen	-	Ovarian cancer (I)	[85]
SVV	-	-	Neuroblstoma (I), rhabdomyosarcoma (I), Neuroendocrine malignancies (I)	[86,87]
Poxvirus	Genetically modified expressing costimulatory and adhesion molecules such as B7-1, LFA-3, ICAM-1	-	Colorectal cancer (I), Melanoma (I)	[88]
PV	-	-	Glioblastoma (I/II)	[89]

HSV-1. Herpes simplex virus-1; ICP. Infected cell protein; GM-CSF. Granulocyte-macrophage colony-stimulating factor; ICI. Immune-checkpoint inhibitor; PD1. Programmed cell death protein 1; CTLA4. cytotoxic T-lymphocyte-associated protein 4; SCC. Squamous cell carcinoma; NDV. Newcastle disease virus; CVA21. Coxsackievirus A21; NSCLC. Nonsmall-cell lung carcinoma; RV. Reovirus; PoV. Poliovirus; AdV. Adenovirus; CRAd. Conditionally replicative adenoviruses; RGD. Arginine-Glycine-Aspartate; CAR. Coxsackievirus and adenovirus receptor; RCC. Renal cell carcinoma; VACV. Vaccinia virus; HCC. Hepatocellular carcinoma; FCU1. Fusion suicide gene; 5-FC. 5-fluorocytosine; 5-FU.5-Fluorouracil; MeV. Measles virus; SVV. Seneca Valley virus; LFA-3. Lymphocyte function-associated antigen-3; ICAM-1. Intercellular adhesion molecule-1; PV. Parvovirus.

**Table 2:** Delivery approaches to enhance tumor access by oncolytic viruses.

Strategy	Approach	Method	Outcome	References
Organic carriers	Stem cell carrier	Mesenchymal Stem cells (Bone marrow, adipose, umbilical cord- menstrual blood)	Off-the-shelf; both systemic and local application; OV shielding; better replication; deliver more viral copies; enhanced tumor tropism; delivery of Ovs to hard-to-access metastatic foci; antiviral immune response evasion; increased persistence of OVs; enhance tumor cell apoptosis; probable toxicity due to trapping mesenchymal stem cells in the lung	[156,157,158]
		Neural Stem cells	Off-the-shelf; improved OV delivery to brain tumors; better response in chemo-resistant ovarian cancer	[159]
	Immune and blood cell carrier	Granulocytes, neutrophils	Delivery to cancer cells located in the bone marrow or spleen; circumvent the problems of systemic delivery; OV shielding	[160,161]
		Dendritic cells	Protect OVs from systemic neutralization, long-term tumor regression; decrease pleural exudation in breast cancer	[162,163]
		T cells	Facilitate systemic OVT in the presence of antiviral nAbs; delivery to cancer cells located in the bone marrow or spleen; Increased efficacy in intratumoral injection; prolonged survival; enhance the efficacy of adoptive cell therapy and OVT; increase selectivity for metastatic tumors; viral concentration in tumor;	[163,164,165]
		Macrophage	Migration to hypoxic tumors; enhanced OV proliferation and antitumor effect in hypoxia; inhibited tumor growth and metastasis; more resistant to antibody neutralization	[166,167]
		Natural killer cell (NK-92 cells transduced with Ad5/37 chimeric fiber)	Strong antitumor effects	[168]
		Cytokine-induced killer cells	Improved tumor trafficking; enhanced antitumor effects	[169]
		Peripheral-blood mononuclear cells	OV shielding from nAb; retained proliferation and selective cytotoxicity for tumor cells, enhanced OV delivery to treat minimal residual disease	[160,170,171]
		Myeloid-derived suppressor cells (MDSC)	Avoid of antiviral responses; preferential migration into tumors; less toxicity following multiple administration; induction of MDSC differentiation towards the M1-like macrophage	[172]
		Platelets	OV shielding from nAb, retained proliferation and cytotoxicity	[160]
		Monocytes	OVs shielding; possibility of multiple administration; more resistant to antibody neutralization	[160,173]
		Erythrocyte, Sickle cell	Improved transfection, high absorption and infection despite nAbs presence	[174,175]
	Carrier Cell lines	HS 578T HeLa A549 MCF-7 CT26 SF268 U937 UR-D7 MC38 MH3924A	Better in vitro manipulation, Trapped in small vessels and decrease circulation, more resistant to antibody neutralization, Iproved viral delivery, replication and intratumoral spread, reduce OV spreading to peripheral organs	[168,176,177,178]
	Other Cells	Blood outgrowth endothelial cells	Shield OVs from nAbs; reduced tumor burden; superior antitumor activity;	[179]
	Extracellular vesicles (EVs)	infected cell-derived EVs, A549-derived EVs, LL/2-derived EVs	increased the transduction and infectious titer; reduced tumor growth; specifically target the tumor; immunological cell death; immune cell infiltration; localized inflammatory effect; provide alternative entry pathways into tumor cells	[180,181]
	Tumor cell membrane	ExtraCRAd (Extra conditionallyreplicating adenoviruses): Membrane of B16.OVA, B16.F10, LL/2, CMT64.OVA, MB49, A549, and SKOV-3 cell lines	OVs wrapped with cancer cell membranes carrying TAA, increased in vitro and in vivo infectivity; control tumor growth with preventive and therapeutic applications; high specific antitumor immune response	[182]
Biomaterials Polymeric carriers	Implant	3D-engineered conformal implant	Constant release of OVs; apoptosis induction; delays tumor recurrence; eradicating post-surgery residual tumors	[183]
	Polymers	Silica, Biosilicification	reduced viral clearance in the liver; evaded nAbs; efficacious anticancer effect; biocompatibility	[184]
		Polylysine-encoded fiber, poly-L-lysine polymer	Better infection capacity	[185,186]
		Lactic-co-glycolic acid nanofiber	Enhanced delivery and therapeutic efficacy; reduced antiviral response	[187]
		multilayer ionic polymer	enhanced oncolytic activity; complement-dependent cytotoxicity; prolonged antitumor activities	[188]
		Alginate	Reduced antiviral response	[189]
		Poly-2-dibutylamino-ethylamine-L-glutamate	High safety and efficacy	[190]
		PolycationsPolybrene	Shielding OVs; bridge virion and cell surface; efficient gene transduction and viral progeny	[191]
		core-cross-linked polyethyleneimine	low immunogenicity and toxicity; higher transduction; stability; improved anticancer cytotoxicity	[192]
		Polyethylene glycol (PEG)ylation, PH-sensitive pegylation	shield virions from nAbs; possibility of dose reduction; increased half-life in circulation	[178,193]
		Poly hydroxypropyl methacrylamide	OVs shielding, increased half-life	[194]
		Polysaccharide	Failed to evade nAbs	[195]
		Silk-elastin-like polymer	OV shielding; better delivery and transduction; higher expression of viral genes; cause acute toxicity	[196]
		Chitosan	OV shielding; enhanced infectivity; induce cell fusion; delay in tumor growth	[197]
		Fibrin and collagen	Sustained release of viral particles	[198]
	Dendrimers	EGFR-targeted dendrimer, Poly-amidoamine dendrimers	selective internalization into EGFR-positive cells; low immunogenicity, toxicity and liver sequestration; Better transduction; OV shielding from nAbs	[199]
	Hydrogel	gelatin-based hydrogel	Decrease antiviral phagocyte response; better DC migration and activation; induction of tumor-specific IFN-γ+ immune cells	[200]
	Scaffolds	Microporous scaffolds	Prevent phagocytosis	[201]
Lipid-based carriers	Liposomes	Anionic liposome, Cationic liposome, Clondrosome (clodronate-loaded liposomes)	Shielding OVs; promoted OV delivery to the cytosol; enhance the tumor cell killing; macrophage depletion and better OV replication; induced expression of antitumorigenic genes	[202,203,204]
	Micelles	Micelles	higher transduction; efficient cellular internalization; improved cancer cell killing; attenuated the host antiviral response; minimal hepatotoxicity; good safety	[205]
Metal-based carriers	Magnetosome	alternating magnetic field (iron oxide) Magnetic nanoparticles magnetically label OV-loaded macrophages and cells OVs labeled with magnetic particle	ECM degradation; enhanced OV uptake; prevention of tumor growth and metastasis; improved targeted therapy; increased tumor macrophage infiltrations; Protection against nAbs,	[206,207,208]
	Metal nanoparticles	Gold nanoparticles	Protected OVs; efficient transduction; enhanced viral cytopathic effect; safe vector for OVs	[209]
Ultrasound		Ultrasound-induced cavitation, Ultrasound + polymers, Ultrasound mediated microbubbles, Ultrasound contrast agents	improve OV extravasation and distribution; kill tumor cells within the ultrasound focal area; retardation of tumor growth; enhanced cell-based OVT	[210,211,212]
Photodynamics	Infrared	Near infrared light (plus gold nanoparticle)	ECM degradation	[213]
	Blue light	Photoactivatable OVs + blue light irradiation	induced replication; no off-tumor toxicity; inhibition subcutaneous tumor growth; therapeutic effect on cancer stem cells	[214]
Pre-treatment	Preconditioning	Granulocyte-macrophage colony-stimulating factor (GM-CSF)	Provide a pool of Potential OV carriers in the circulation: monocyte, macrophage, granulocytes, MDSCs, and CD11b+ cells; enhanced viral delivery; protected OVs from nAb	[215]
Targeting ligands	Nanoparticle natural and engineered ligands	BiTEs, Trispecific Abs, Arginine-glycine-aspartic acid motif (RGD), Glycoprotein B/C, Neurotensin, Folic acid, Trastuzumab, Cetuximab, VEGF/bFGF, Biotin-EGF, CD71 and CD62E/P- immunovirosomes	Bridge tumor cells and OVs/immune cells; OV release in hypoxic/acidic TME; better cell entry; enhanced tumor tropism, nAb evasion; prolonged blood retention time; improved transduction;	[15,157,178,216,217,218]
	Viral particles	extracellular enveloped viral particle	Rapid OV spread within the TME; prevent removal by immune response; well adapted for systemic infusion;	[219]
Pharmacologic manipulation	Systemic and local pharmacotherapy	Angiotensin receptor blocker, paclitaxel, nitric oxide, nitroglycerin, bradykinin, Histamine TH-302 and PR-104 IC87114 or idelalisib (PI3Kδ-inhibitor)	activated local matrix metalloproteinases to disrupt the ECM; temporal vasodilation and better perfusion; OV activation in hypoxia; potentiateintravenous delivery of OV	[178,211,220,221]
		Cobra venom factor (CP40)	Complement inhibition; increase in OV titer in the blood; Prolonged OV existence	[222]
		Cyclophosphamide, Rapamycin	enhanced OV replication and activity; Avoid antiviral immune response	[223]
		Polyinosinic acide	Saturate scavenger receptors; prevent OV sequestration by Kupffer cells (liver macrophages); requirement of low dose OVs; lower toxicity; improve transduction	[224]
Intratumoral spread of OVs	ECM-degradation	Hyaloronidase, Decorin, Relaxin, Chondroitinase, Matrix metalloproteinases, Collagenase, Bromelain, TAF depletion, LOX inhibition antibodies	Enhanced intratumoral spread of OVs; decrease matrix crosslinking and deposition	[6,211,225,226]
	Cellular junction opener	penton-dodecahedra, Junction Opener-1	Enhanced intratumoral spread of OVs	[226]
	Fusogenic proteins	Natural or engineered fusogenic OVs: MeV, NDV, RV, SeV, MuV, RSV, GALV, PoxV, VACV, VSV, HSV, and AdV	Improved infection; Enhanced tumor killing capability	[226,227,228,229,230,231,232]
	Vasculature degradation agents	Trombospondin-1 (TSP-1) TSP-1 peptide 3TSR Endostatin Anti-VEGF scAb	Better perfusion and delivery; enhanced intratumoral spread of OVs; Tumor necrosis; reduced hypoxia	[6,233]

OV. Oncolytic virus; OVT. OV therapy; ECM. Extracellular matrix; BiTE. Bispecific T cell engager; VEGF. Vascular-endothelial growth factor; bFGF. basic fibroblast GF; PI3K. Phosphoinositide 3-kinase; TAF. Tumor-associated fibroblast; Lox. Lipoxygenase.
